# The Tol-Pal System Plays an Important Role in Maintaining Cell Integrity During Elongation in *Escherichia coli*

**DOI:** 10.3389/fmicb.2022.891926

**Published:** 2022-05-03

**Authors:** Sohee Park, Hongbaek Cho

**Affiliations:** Department of Biological Sciences, College of Natural Sciences, Sungkyunkwan University, Suwon, South Korea

**Keywords:** gram-negative bacteria, beta lactam, the Rod system, cell wall, elongation, Tol-Pal, class A PBP, Lpo factor

## Abstract

The Tol-Pal system is a transenvelope complex widely conserved among Gram-negative bacteria. It is recruited to the septal ring during cytokinesis, and its inactivation causes pleiotropic phenotypes mainly associated with the division process. From our genetic screen to identify factors required for delaying lysis upon treatment of beta lactams, we discovered that the *tol-pal* mutant shares similar defects with mutants of the major class A PBP system (PBP1b-LpoB) in terms of lysis prevention. Further phenotypic analyses revealed that the Tol-Pal system plays an important role in maintaining cell integrity not only during septation, but also during cell elongation. Simultaneous inactivation of the Tol-Pal system and the PBP1b-LpoB system leads to lysis during cell elongation as well as during division. Moreover, production of the Lpo activator-bypass PBP1b, but not wild-type PBP1b, partially suppressed the Tol-Pal defect in maintaining cell integrity upon treatment of mecillinam specific for the Rod system, suggesting that the Tol-Pal system is likely to be involved in the activation of aPBP by Lpo factors. Overall, our results indicate that the Tol-Pal system plays an important role in maintaining cell wall integrity during elongation in addition to its multifaceted roles during cytokinesis.

## Introduction

Bacterial cells are surrounded by a giant mesh-like peptidoglycan (PG) structure outside the cytoplasmic membrane, which functions as a bacterial cell wall that maintains cell shape and provides protection from osmotic rupture. PG consists of beta-1,4-linked glycan polymers of alternating *N*-acetylglucosamine (GlcNAc) and *N*-acetylmuramic acid (MurNAc) that are crosslinked by short peptides (H‘`oltje, 1998; Vollmer et al., 2008). Thus, PG assembly requires PG glycosyltransferases (PGTs) that polymerize the glycan strands and transpeptidases (TP) that crosslink the peptides attached to the PG glycans.

A major class of PG synthetic enzymes are high molecular weight penicillin-binding proteins (PBPs) that were identified based on their ability to covalently attach to penicillin at the TP active site (Sauvage et al., 2008). The synthetic PBPs are classified into two groups based on their enzymatic activities. Enzymes known as class A PBPs (aPBPs) have the PGT domain in addition to the TP domain. In Gram-negative bacteria, the aPBPs in the inner membrane (IM) need activation from their cognate Lpo factors located in the outer membrane (OM) to function efficiently as PG synthases. Two pairs of aPBPs and Lpo factors, PBP1a-LpoA and PBP1b-LpoB, function in parallel and show synthetic lethality in *E. coli* (Paradis-Bleau et al., 2010; Typas et al., 2010). The other group of synthetic enzymes known as class B PBPs (bPBPs) show only TP activity. The bPBPs function in conjunction with SEDS (shape, elongation, division, and sporulation) family proteins that provide PGT activity (Cho et al., 2016; Meeske et al., 2016; Sjodt et al., 2018; Reichmann et al., 2019; Taguchi et al., 2019). SEDS-bPBP pairs function as integral PG synthases of the cell wall assembly complexes whose assembly is coordinated by cytoskeletal elements.

In rod-shaped bacteria such as *E. coli*, the cell wall is elongated by the Rod system (elongasome), a multiprotein complex whose assembly is coordinated by an actin-like protein MreB (Zhao et al., 2017; Rohs and Bernhardt, 2021). A SEDS-bPBP pair called RodA-PBP2 functions as the PG synthase of the Rod system. After elongation, the cell is divided into two daughter cells by activity of the divisome. Formation of the divisome begins with the assembly of a tubulin homolog FtsZ into a ring-like structure underneath the cytoplasmic membrane at the future division sites. The FtsZ ring then recruits dozens of different proteins to form the divisome or septal ring, in which another SEDS-bPBP pair, FtsW and PBP3, functions as the integral PG synthetic enzyme (Taguchi et al., 2019; Rohs and Bernhardt, 2021). A mature divisome carries out cell wall constriction against the turgor pressure of the cytoplasm. In addition, extensive PG remodeling occurs along with PG synthesis during division to separate the newly synthesized cell wall at the division site and generate the new cell poles.

In Gram-negative bacteria, completion of cytokinesis also requires the invagination of the OM at the division sites, for which the Tol-Pal system is thought to play a critical role (Gerding et al., 2007; Szczepaniak et al., 2020a). The Tol-Pal system has five core components: three IM proteins TolQ, TolR, and TolA, a periplasmic protein TolB, and an OM lipoprotein Pal that localize to the division sites. Inactivation of any of these components has been reported to cause pleiotropic phenotypes mostly associated with cytokinesis, such as OM permeability defects, production of OM vesicles from the division sites, delayed OM constriction during division, and septal PG splitting defects (Gerding et al., 2007; Egan, 2018; Szczepaniak et al., 2020b; Yakhnina and Bernhardt, 2020). In *E. coli*, there are two additional genes at the *tol-pal* locus, *ybgC* and *cpoB*. Among these, *ybgC* encodes a cytoplasmic acyl CoA thioesterase that was proposed to function in regulation of cell motility but does not show clear connection to the Tol-Pal function (Gao et al., 2017). *cpoB* encodes a periplasmic protein that interacts with TolA (Krachler et al., 2010). CpoB was shown to interact with PBP1b to negatively control the TP activity of PBP1b (Gray et al., 2015), but its inactivation did not cause the characteristic Tol-Pal phenotype (Szczepaniak et al., 2020b).

In this study, we aimed to identify and characterize factors involved in maintaining cell integrity upon treatment of beta lactam antibiotics. Beta lactams inhibit TP activity of the PBPs and TP inhibition prevents the crosslinking of newly synthesized PG material to the existing matrix, eventually causing bacterial lysis. However, how bacteria cope with TP inhibition to avoid or delay beta lactam-induced lysis has not been well understood. Poor characterization of the processes that function in maintaining cell integrity after TP inhibition is partly due to the lack of a genetic system to identify factors directly involved in lysis prevention. Genetic selections with beta lactam drugs have been instrumental in revealing genes involved in beta-lactam resistance and PG assembly (Vinella et al., 1992; Cao et al., 2004; Sobel et al., 2005; Cho et al., 2014; Lai et al., 2017). Yet, these selections showed limited success in identifying factors directly involved in lysis prevention because the lethal mechanism of beta lactams is complex and prevention of lysis often does not lead to survival upon treatment of these drugs (Moreillon et al., 1990; Cho et al., 2014). Thus, a genetic screen that does not depend on the survival of mutant cells is necessary for studying the mechanisms that counteract the onset of lysis upon treatment of beta lactams.

To circumvent the limitation of genetic selections with beta lactam drugs, we used a transposon-sequencing (Tn-seq)-based screen to identify mutations that accelerate lysis after beta lactam treatment. Rather than selecting for survivors, we collected unlysed cells of a transposon mutant library after brief drug treatment. Mutations that affected the lysis kinetics were then identified by comparing the transposon insertion profiles between the unlysed cells of the beta lactam-treated library and the cells of the library grown without drug treatment. The Tol-Pal system genes were identified among the genes important for maintenance of cell integrity upon treatment of bPBP-specific beta lactams. Interestingly, the *tol-pal* genes were not only important for maintaining cell integrity upon inhibition of TP activity of the divisome, but also upon inhibition of TP activity of the Rod system. Further analyses of cell elongation phenotypes of the *tol-pal* mutant suggested that the Tol-Pal system is required for efficient PG synthesis by class A PBPs during cell elongation. Thus, our study indicates that the Tol-Pal system is involved in maintenance of cell envelope integrity during elongation in addition to its roles related to various aspects of cell division.

## Materials and Methods

### Bacterial Strains and Growth Conditions

Strains and plasmids used in this study are listed in the Supplementary Tables 1, 2. All *E. coli* strains used in the reported experiments are derivatives of MG1655 (Guyer et al., 1981). Bacterial cells were grown in lysogeny broth (LB) [1% tryptone, 0.5% yeast extract, 0.5% NaCl] or minimal M9 medium supplemented with 0.2% casamino acids and 0.2% glucose. Antibiotics were used at 25 μg/ml (chloramphenicol; Cm), 37.5 μg/ml (kanamycin; Kan) or 5 μg/ml (tetracycline; tet).

### Strain Construction

Gene deletion mutants were constructed to resemble those in the Keio knockout collection (Baba et al., 2006). The Kan*^R^* cassette was amplified by using pKD13 as a template with sequences homologous to the 5′ and 3′ ends of the target genes in the chromosome. The sequences of the primer pairs used for gene deletion are listed in Supplementary Table 3. The amplified DNA was electroporated into MG1655 harboring either pKD46 or pBO51 that expresses the lambda Red genes from an arabinose-inducible promoter (pKD46) or high temperature-inducible promoter (pBO51), and recombinants were selected on LB agar supplemented with 37.5 μg/ml kanamycin (Johnson et al., 2004).

Strains with multiple deletion mutations were made by sequential introduction of each deletion *via* P1 transduction followed by removal of the *Kan^R^* cassette using FLP recombinase expressed from pCP20, leaving a *frt* scar sequence at each deletion locus (Datsenko and Wanner, 2000). The correct orientation of the DNA flanking *frt* sequences in multiple deletion mutants was confirmed for all deletions in each mutant.

### Generation of the Transposon Insertion Library

MG1655 was mutagenized with the Ez-Tn5 < Kan-2 > transposome (Epicenter) as previously described (Bernhardt and Boer, 2004). Mutants were selected on agar for kanamycin resistance at 30°C, yielding libraries consisting of approximately 4 × 10^5^ independent transposon insertions. The mutant library was harvested by scraping colonies from the agar surface and suspending them in LB broth. The suspension of the mutant library was mixed with glycerol to a final concentration of 15%, aliquoted, and kept frozen at −80°C.

### Separation of Unlysed Cells After Beta Lactam Treatment

A transposon mutant library of MG1655 was thawed and incubated in LB for roughly two doublings. The resuscitated mutant library culture was diluted to an OD_600_ of 0.04 in 25 mL LB lacking or supplemented with 0.1 μg/ml aztreonam or 2.5 μg/ml mecillinam and incubated for 2 h at 30°C with agitation (250 rpm). After incubation, the cells of each culture were pelleted, washed with 1X PBS, and resuspended in 0.5 mL DNase I solution [0.25 mg DNase I (Roche) in 10 mM Tris–HCl, pH 7.5, 2.5 mM MgCl_2_, 0.1 mM CaCl_2_]. The cell suspensions in DNase I solution were incubated for 10 min at 37°C to remove extracellular DNA released from lysed cells. DNase I-treated cells were pelleted, resuspended in 1X PBS supplemented with 10 mM EDTA, and incubated for 10 min at 80°C to inactivate DNase I. The cells were pelleted and kept frozen at −80°C until preparation of genomic DNA from the unlysed cells.

### Transposon Insertion Site Sequencing

Genomic DNA from each cell pellet was extracted, fragmented, and poly-C tailed as previously described (Lai et al., 2017). The transposon-chromosome junctions in the resulting DNA were amplified by using Easy-A Hi-Fi Cloning System (Agilent Technologies). The primers used were the poly-C tail-specific primer 5′- GTGACTGGAGTTCAGACGTGTGCTCTTCCGAT CTGGGGGGGGGGGGGGGG-3′ and the transposon-specific primer 5′-ACCTGCAGGCATGCAAGCTTCAGGG-3′. The transposon-chromosome junctions were further amplified in a second nested PCR with primers that add sequencing barcodes to each mutant library, NEBNext Multiplex Oligos for Illumina (NEB) and the transposon-specific primer 5′- AATGATACGGCGACCACCGAGATCTACACTCTTTTCAGG GTTGAGATGTGTATAAGAGA-3′. The final PCR products were quantified and equal amounts of each barcoded library were mixed. The pooled sequencing library was run on a 2% agarose gel, and DNA fragments ranging from 200 and 500 bp were excised and purified using QIAquick Gel Extraction Kit (Qiagen). The resulting library was sequenced using a MiSeq reagent kit V3 (150-cycle) (Illumina) with the custom primer 5′-ACACTCTTTTCAGGGTTGAGATGTGTATAAGAGACAG-3′. Sequencing reads were trimmed using trimmomatic (Bolger et al., 2014) to remove adaptor sequences, and mapped to the *E. coli* MG1655 genome (NC_000913) using bowtie 1.0.0 (Langmead et al., 2009). Differences in the total number of reads at any given transposon insertion site between untreated and beta lactam-treated samples were determined using a Mann–Whitney *U* test. Transposon insertion profiles were visualized using the Sanger Artemis Genome Browser and Annotation tool.

### Bocillin-Binding Assays

Cultures of MG1655 and SP10 (Δ*tol-pal*) strains were grown to an exponential phase (OD_600_ between 0.4 and 0.5) in LB at 30°C. Aztreonam or mecillinam was added to 15 mL cultures at the final concentrations of 0, 0.1, 1, or 10 μg/ml and incubated for 5 min at 30°C. Cells were then collected by centrifugation at 4°C, washed with ice-cold 1X PBS twice, resuspended in 500 μl 1X PBS supplemented with 10 mM EDTA and 15 μM Bocillin (Invitrogen), and incubated at room temperature for 15 min. After incubation, the cell suspensions were washed with 1X PBS once, resuspended in 500 μl 1X PBS, and disrupted by sonication. After a brief spin for 1 min at 4 Krcf to remove undisrupted cells, membrane fractions were pelleted by ultracentrifugation at 200 Krcf for 20 min at 4°C. The membrane fractions were then washed with 1X PBS and resuspended in 50 μl 1X PBS. Resuspended samples were mixed with 50 μl 2X Laemmli sample buffer and boiled for 10 min at 95°C. After measuring the total protein concentrations of each sample with the NI-protein assay (G-Biosciences), 25 μg of total protein for each sample was then separated on a 4–20% gradient SDS–PAGE gel and the labeled proteins were visualized using a Typhoon 9400 fluorescence imager (GE Healthcare) with excitation at 488 nm and emission at 520 ñm.

### Microscopy

Growth conditions prior to microscopy are described in the figure legends. Prior to imaging, cells were immobilized on 2% agarose pads containing 1X M9 salts and covered with #1.5 coverslips. Micrographs were obtained using a Leica DM2500 LED microscope equipped with a Leica DFC7000 GT camera, Fluo Illuminator LRF 4/22, HC PL APO 100x/1.40 Oil Ph3 objective lens, and Leica Las X acquisition software. Images in the green channel were obtained using an L5 filter cube.

## Results

### Identification of Mutations That Accelerate Lysis Upon Beta Lactam Treatment

**FIGURE 1:**
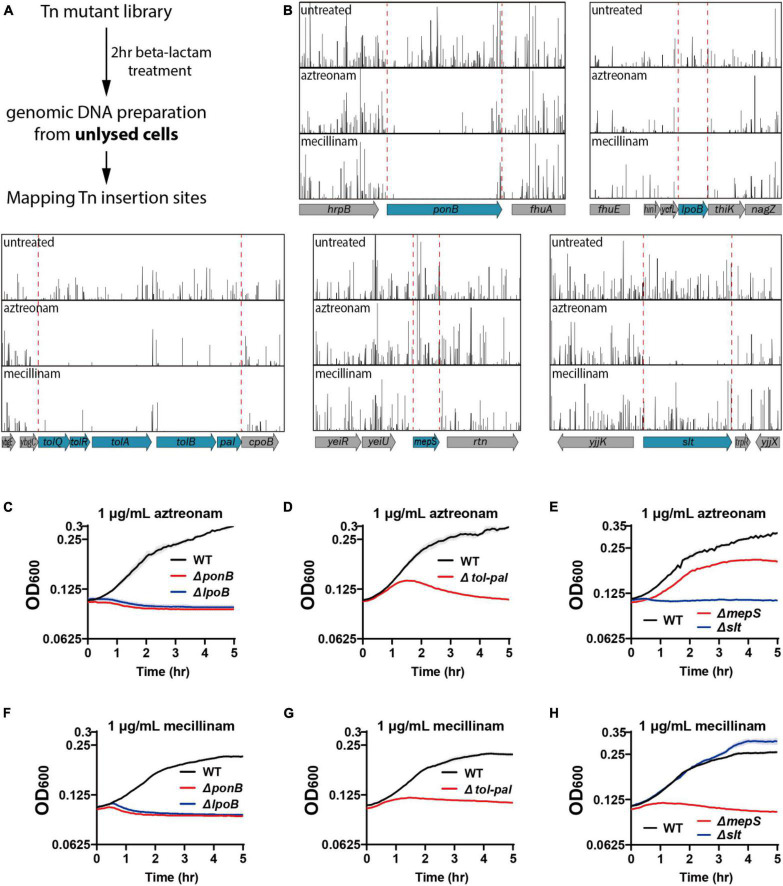
Tn-seq-based screening of genes important for maintaining cell integrity. **(A)** Scheme of the transposon screen used to identify genes required for maintaining cell integrity upon beta lactam treatment. Cultures of an *E. coli* transposon mutant library were treated for 2 h with aztreonam or mecillinam that specifically target PBP3 or PBP2, respectively. Unlysed cells were collected after beta lactam treatment and the transposon insertion profiles were compared with that of the untreated library to identify mutations that cause rapid lysis upon beta lactam treatment. **(B)** Transposon insertion profiles of genomic regions near the genes that showed significant reduction in transposon insertion frequency. Each vertical line represents a sequenced insertion site and the heights of the lines represent the number of reads mapped at those sites. **(C–H)** Lysis curves of the mutant strains identified from the transposon screen. MG1655, WJ2 (Δ*ponB*), SP38 (Δ*lpoB*), SP10 (Δ*tol-pal*), SP8 (Δ*mepS*), and SP178 (Δ*slt*) strains were grown overnight in LB at 30°C. The overnight cultures were diluted to an OD_600_ of 0.02 in LB and grown to an OD_600_ between 0.2 and 0.3. The exponential cultures were diluted to an OD_600_ of 0.1 in LB containing either aztreonam **(C–E)** or mecillinam **(F–H)** at a final concentration of 1 μg/ml and grown at 30°C. The optical density of the cultures was measured in triplicate in a 96-well microtiter plate for 5 h with agitation at 30°C after beta lactam treatment. The solid lines represent the means and the shades standard deviations.

In a transposon screen to identify factors important for maintaining cell integrity upon beta lactam treatment, we used mecillinam and aztreonam that bind specifically to PBP2 of the Rod system and PBP3 of the divisome, respectively (Curtis et al., 1979; Kocaoglu and Carlson, 2015). These drugs were chosen because phenotypic analyses would be easier with beta-lactams that mainly target a single PBP. We also reasoned that it would be advantageous for identification of mutations that accelerate lysis because bPBP-specific beta-lactams result in slower lysis than drugs that bind to multiple PBPs (Satta et al., 1995). For the actual screen, wild-type *E. coli* MG1655 cells were mutagenized with the EZTn5-Kan2 transposome to generate a mutant library consisting of approximately 4 × 10^5^ independent insertions. The mutant library was grown in LB to the exponential phase (OD_600_ = 0.4), diluted in LB lacking or containing beta-lactams, and incubated for 2 hrs. After drug treatment, genomic DNA was isolated from the unlysed cells of each sample and the transposon insertion sites were mapped by high-throughput sequencing (Figure 1A). Comparison of the transposon insertion profiles between each sample revealed that transposon insertion in several genes significantly decreased in the sample collected after drug treatment relative to the sample prepared without drug treatment (Figure 1B). The decrease in transposon insertion densities indicated that the mutants bearing the transposon insertion lysed much faster than other mutants in the library upon beta-lactam treatment.

**FIGURE 2:**
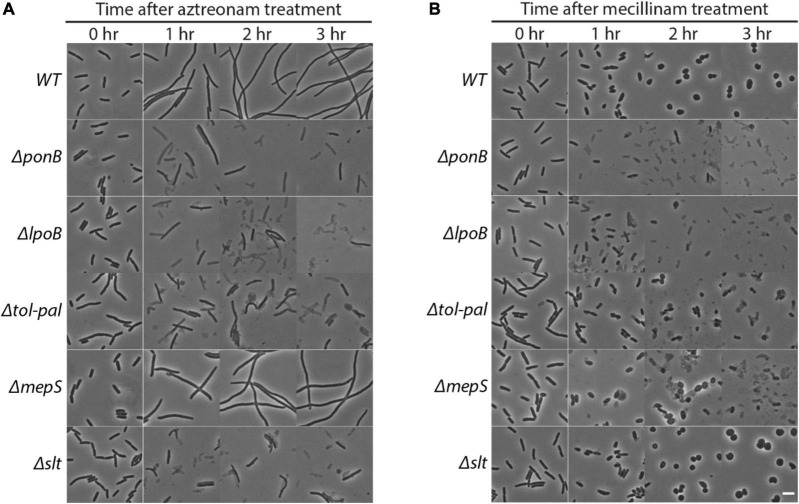
Effects of *ponB*, *lpoB*, *tol-pal*, *mepS* or *slt* mutations on morphology and lysis phenotypes upon beta lactam treatment. The strains used in Figure 1 were grown and diluted in LB supplemented with 1 μg/ml aztreonam **(A)** or 1 μg/ml mecillinam **(B)** using the same procedure described for Figure 1. The cultures were then incubated in test tubes at 30°C with agitation. The cell shape and lysis phenotype of cells in each culture were observed before and at 1–3 h after beta lactam treatment using phase contrast optics. The scale bar represents 5 μm.

The genes that showed a clear reduction in transposon insertion density after beta-lactam treatment were *ponB, lpoB*, *tolQ-pal, mepS*, and *slt* (Figure 1B). Among these genes, *ponB, lpoB*, and *tolQ-pal* showed reduction regardless of the drug used. On the other hand, *mepS* and *slt* showed beta lactam-specific reduction: *mepS* upon mecillinam treatment and *slt* upon aztreonam treatment. To validate the hits identified from the transposon insertion site sequencing (Tn-seq) experiment, we made null mutants of the identified genes and compared the lysis kinetics and shape change of the mutant strains with the wild-type strain after drug treatment (Figures 1C–H, 2A-B and Supplementary Figure 1). Each mutant showed lysis phenotypes consistent with the Tn-seq results. The Δ*ponB*,Δ*lpoB*, Δ*tolQ*, Δ*tolR*, Δ*tolA*, Δ*tolB*, and Δ*pal* strains lysed faster than the wild-type strain upon either mecillinam or aztreonam treatment. The Δ*mepS* strain lysed faster upon mecillinam treatment, but not much upon aztreonam treatment. In contrast, the Δ*slt* strain showed accelerated lysis upon aztreonam treatment, but not upon mecillinam treatment.

### The PBP1b-LpoB Pair, but Not the PBP1a-LpoA Pair, Is Important for Maintaining Cell Wall Integrity During Elongation and Division

While transposon insertions in *ponB* and *lpoB* genes encoding PBP1b and LpoB decreased dramatically upon treatment of mecillinam or aztreonam, transposon insertions in *ponA* and *lpoA* genes encoding another aPBP-Lpo factor pair did not decrease noticeably upon treatment of either beta lactam drug (Supplementary Figure 2A). Although *E. coli* cells need only one of these aPBP-Lpo systems for survival under normal laboratory growth conditions, it has been observed that inactivation of the PBP1b-LpoB system causes a severe defect in maintaining cell integrity under various cell wall-perturbing conditions (del Portillo and de Pedro, 1990, 1991; Paradis-Bleau et al., 2010; Typas et al., 2010; Vigouroux et al., 2020). In addition, an elevated lysis phenotype of the *ponB* and *lpoB* mutants under normal growth conditions was also identified in a genetic screen with CPRG (Paradis-Bleau et al., 2014). In contrast, the *ponA* and *lpoA* mutants displayed only minor phenotypes except for the synthetic lethality with *ponB-lpoB* mutations (Yousif et al., 1985; Mueller et al., 2019; Vigouroux et al., 2020). Consistent with the previous reports and our Tn-seq results, the Δ*ponA* and Δ*lpoA* strains did not show accelerated lysis upon treatment of mecillinam or aztreonam when we examined the lysis kinetics of these strains upon treatment of either beta lactam (Supplementary Figures 2B,C). These results indicated that PBP1b-LpoB pair plays a major role in maintenance of cell wall integrity upon inhibition of TP activity of the Rod system or the divisome, but PBP1A-LpoA function is dispensable under the same circumstances.

### Outer Membrane Permeability Defects Do Not Account for the Accelerated Lysis of the Tol-Pal Mutants Upon Beta-Lactam Treatment

**FIGURE 3:**
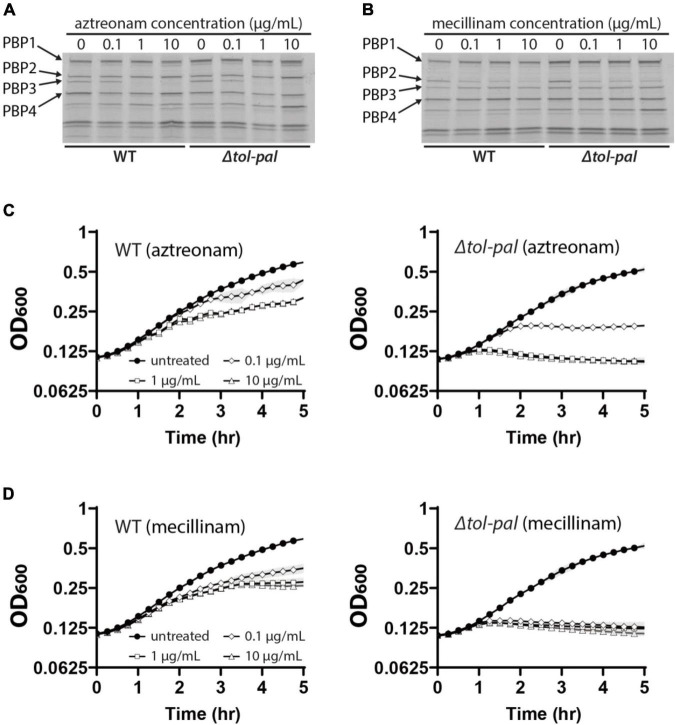
OM permeability defects do not account for the accelerated lysis of the *tol-pal* mutant. **(A,B)** Bocillin assay to compare the binding specificity of aztreonam and mecillinam to PBPs between the wild type and Δ*tol-pal* strains. Cultures of MG1655 and SP23 (Δ*tol-pal*) strains were grown to the exponential phase (OD_600_ = 0.5) in LB at 30°C and treated with the indicated concentrations of aztreonam **(A)** or mecillinam **(B)** for 5 min. Cells harvested from 15 ml of each culture were labeled with Bocillin. Bocillin-labeled PBPs of each sample were separated on 4–20% SDS-PAGE gels and visualized by fluorescence scanning. **(C,D)** Lysis curves of MG1655 and SP23 strains after treatment of the cultures with the indicated concentrations of aztreonam **(C)** or mecillinam **(D)**. MG1655 and SP23 cells were grown to the exponential phase (OD_600_ between 0.2 and 0.3) as described for Figure 1, and diluted to an OD_600_ of 0.1 in LB supplemented with the indicated final concentrations of aztreonam **(C)** or mecillinam **(D)**. The optical density of the cultures was measured in the same way as in Figure 1.

Accelerated lysis of the *tol-pal* mutant upon treatment of aztreonam or mecillinam suggested that the Tol-Pal system plays an important role in maintaining the integrity of the cell wall. However, because the *tol-pal* mutant has a permeabilized OM (Cascales et al., 2000; Lloubès et al., 2001), it also seemed possible that the OM permeability defect might be responsible for the accelerated lysis of the *tol-pal* mutant upon beta-lactam treatment. Beta-lactams might accumulate to a higher concentration in the periplasm of the *tol-pal* mutant than the wild-type strain due to the OM permeability defect, which might in turn cause non-specific inhibition of other PBPs such as aPBPs that are important for maintaining cell integrity. Thus, we examined if the OM permeability defect of the Δ*tol-pal* strain affected the PBP-binding profile of aztreonam and mecillinam by using a Bocillin assay. A noticeable difference was not observed between the wild type and Δ*tol-pal* strains across a broad range of concentrations (Figures 3A,B), indicating that the permeability defect of the Δ*tol-pal* strain does not significantly affect the PBP-binding specificity of mecillinam and aztreonam. Furthermore, the Δ*tol-pal* strain lysed faster than the wild-type strain even when treated with 100-fold less amounts of aztreonam or mecillinam than the wild-type strain (Figures 3C,D). These results suggested that the permeability defect is not likely to be responsible for the accelerated lysis of the Δ*tol-pal* strain upon treatment of aztreonam or mecillinam, and the Tol-Pal system likely plays a more direct role in maintaining cell integrity.

### Accelerated Lysis of the Tol-Pal Mutant Upon Mecillinam Treatment Cannot Be Suppressed by Inhibiting Division

The accelerated lysis of the Δ*tol-pal* strain upon mecillinam treatment was especially interesting because the Tol-Pal system localizes at the division sites and most of the reported phenotypes of the *tol-pal* mutant are related to the division process (Gerding et al., 2007; Egan, 2018; Szczepaniak et al., 2020b). Upon mecillinam treatment, wild-type *E. coli* forms spherical cells that become larger and eventually lyse (Spratt, 1975). However, mecillinam-treated *tol-pal* mutant cells lysed early before forming spherical cells, indicating that the *tol-pal* mutant has a severe defect in maintaining cell integrity upon inhibition of the Rod system (Figure 2B). Because most known functions of the Tol-Pal system are related to cell division, we wondered if the *tol-pal* mutant somehow lysed at the division sites upon mecillinam treatment even though mecillinam targets the Rod system responsible for cell elongation.

**FIGURE 4:**
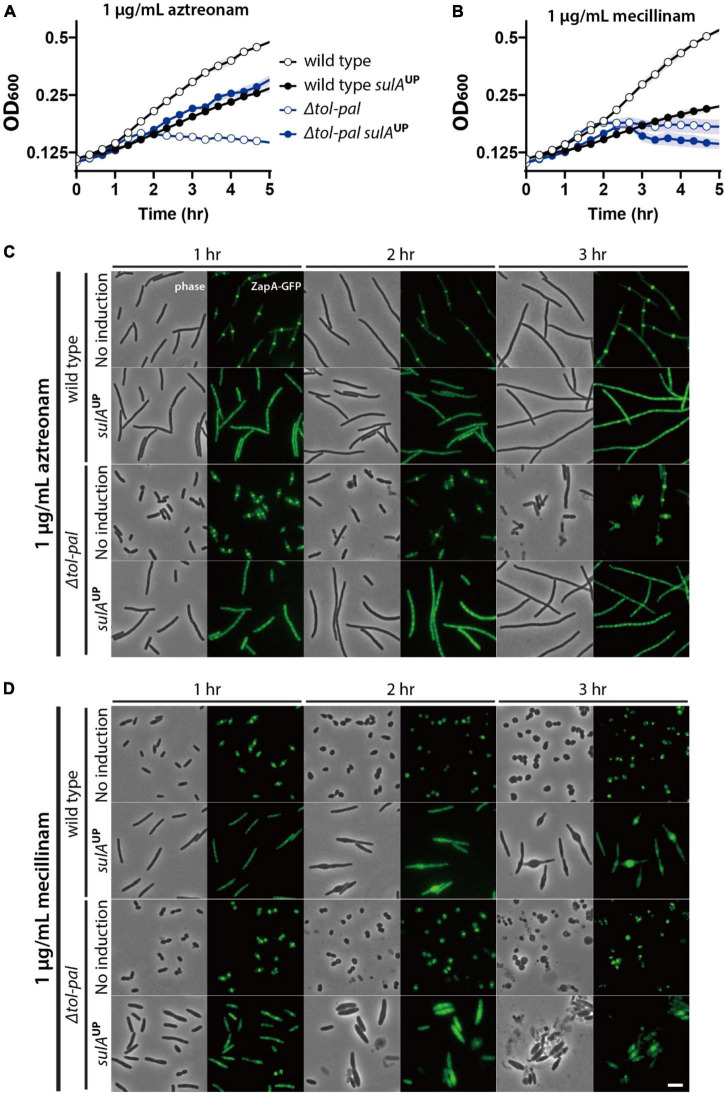
Accelerated lysis of the *tol-pal* mutant upon mecillinam treatment is not suppressed by inhibiting division. **(A,B)** SP97 (*zapA-gfp*) and SP98 (Δ*tol-pal*, *zapA-gfp*) strains harboring pSP6 (P_*tac*_:*sulA*) were grown overnight in M9 minimal medium supplemented with 0.2% glucose, 0.2% casamino acids (CAA), and 5 μg/ml tetracycline at 30°C. The overnight cultures were diluted to an OD_600_ of 0.02 in the same medium and grown to an OD_600_ between 0.2 and 0.3. Then, cells were washed and diluted in M9-glucose-CAA medium lacking or supplemented with 1 mM IPTG for induction of *sulA* expression. After induction for 30 min, the cultures were diluted to an OD_600_ of 0.1 in the same corresponding media containing either aztreonam **(A)** or mecillinam **(B)** at a final concentration of 1 μg/ml. The optical density of the cultures was measured in the same way described for Figure 1. **(C,D)** The same cultures were incubated in test tubes at 30°C with agitation after dilution in media containing aztreonam **(C)** or mecillinam **(D)**. The cell shape and lysis phenotype of the cells in each culture were observed using phase contrast optics at 1–3 h after beta lactam treatment. The effects of *sulA* induction on division inhibition of the same cells was monitored using ZapA-GFP as a marker for divisome assembly. The discrete bands of fluorescence in the absence of *sulA* induction represents the assembled divisomes, while diffuse fluorescence signal upon induction of *sulA* expression indicates inhibition of divisome assembly. The scale bar represents 5 μm.

To examine this possibility, we tested if inhibition of divisome assembly suppresses the accelerated lysis of the *tol-pal* mutant upon treatment of beta lactams. Assembly of the divisome was inhibited by overexpressing the FtsZ antagonist SulA, and beta lactams were added after confirming the inhibition of divisome assembly by visualizing the Z-ring with the *zapA-gfp* fusion expressed from the native *zapA* genomic locus. The effect of division inhibition was tested in M9 minimal medium because we found that *sulA* overexpression somehow enhanced the lysis of mecillinam-treated wild type cells when the cultures were grown in LB. As expected, accelerated lysis of the *tol-pal* mutant upon aztreonam treatment was almost completely suppressed by inhibition of division, consistent with the idea that lysis upon aztreonam treatment occurs at the division sites (Figure 4). However, the accelerated lysis of the *tol-pal* mutant upon mecillinam treatment was not suppressed by *sulA* overexpression, suggesting that the cell integrity defect of the *tol-pal* mutant is not restricted to the division sites (Figure 4).

### The Tol-Pal System Plays a Role in Maintaining Cell Integrity at Locations Other Than the Division Sites

Ineffectiveness of divisome inhibition in suppressing the accelerated lysis of the *tol-pal* mutant upon mecillinam treatment suggested that the Tol-Pal system plays a role in maintaining cell integrity not only in the dividing cells but also in the elongating cells. Thus, we examined another lysis phenotype related to the *tol-pal* mutation to gauge if the Tol-Pal system is involved in maintaining cell integrity at locations other than the division sites. Mutation of the *tol-pal* genes has been shown to cause a synthetic lysis phenotype with inactivation of the PBP1b-LpoB system (Typas et al., 2010; Markovski et al., 2016). It was assumed that this synthetic lysis would occur mostly at the division sites because the Tol-Pal system was known to function mainly at the division sites and the PBP1b-LpoB system activity was also thought to be important for maintaining cell integrity at the division sites (Typas et al., 2010). We reasoned that this synthetic lysis would also occur during cell elongation if the Tol-Pal system plays an important role in maintaining cell integrity during elongation. If so, the synthetic lysis caused by inactivation of both the Tol-Pal system and the PBP1b-LpoB system would not be efficiently suppressed by division inhibition.

**FIGURE 5:**
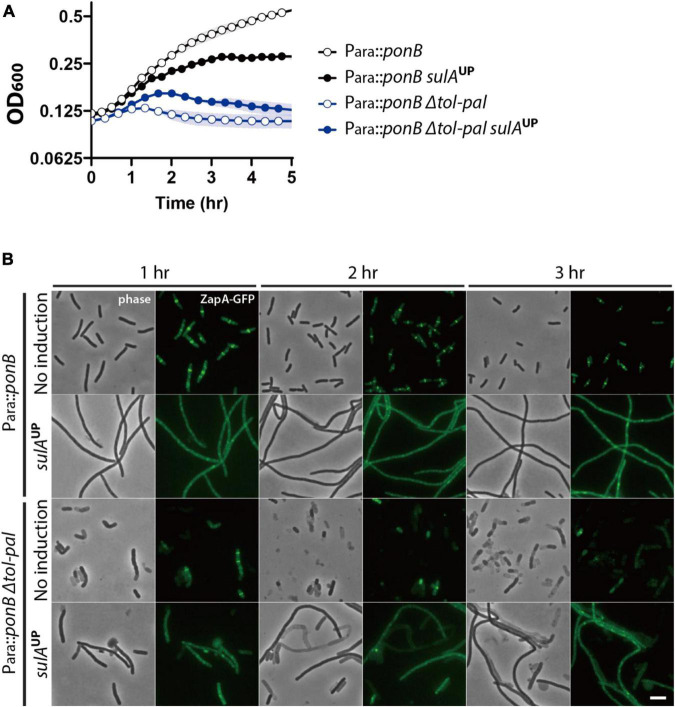
Lysis induced by *ponB* depletion in the *tol-pal* mutant is only partially suppressed by division inhibition. **(A)** SP199 (P_*ara*_:*ponB*, *zapA-gfp*) and SP200 (P_*ara*_:*ponB*, Δ*tol-pal*, *zapA-gfp*) strains harboring pSP6 (P_*tac*_:*sulA*) were grown overnight in LB supplemented with 0.2% arabinose and 5 μg/ml tetracycline at 37°C. The overnight cultures were washed, diluted in LB supplemented with 5 μg/ml tetracycline but lacking arabinose to an OD_600_ of 0.02, and grown for 2 h to an OD_600_ between 0.2 and 0.3 at 37°C to deplete *ponB* expression. Then, the cultures were washed in no salt LB, and diluted to an OD600 of 0.1 in no salt LB lacking or supplemented with 1 mM IPTG for induction of *sulA* expression. The optical density of the cultures was measured in the same way as in Figure 1 except that the culture plate was incubated at 37°C in the plate reader. **(B)** The same cultures in no salt LB lacking or supplemented with 1 mM IPTG were grown in test tubes at 37°C for visualization of the lysis phenotype of each strain. The cells in the cultures were imaged using phase contrast and GFP optics at 1–3 h after dilution in no salt LB to monitor lysis and divisome assembly. The scale bar represents 5 μm.

To examine the effect of division inhibition on the synthetic lysis phenotype, we constructed the strain SP200 [P_*ara*_:*ponB*, Δ*tol-pal*, *zapA-gfp*] in which the *tol-pal* locus is deleted and PBP1b can be depleted by growth in medium lacking arabinose. SP200 lysed upon depletion of PBP1b when it was grown in no salt LB. This synthetic lysis would be efficiently suppressed by division inhibition if the Tol-Pal system is mainly involved in maintaining cell integrity at the division sites. On the other hand, the synthetic lysis would only be partially suppressed by division inhibition if Tol-Pal system activity is also required for cell elongation. The synthetic lysis was not suppressed efficiently by *sulA* overexpression, indicating that the synthetic lysis occurs not only at the division sites but also during elongation (Figure 5).

### Inactivation of the PBP1a-LpoA System Also Aggravates the Tol-Pal Defect

**FIGURE 6:**
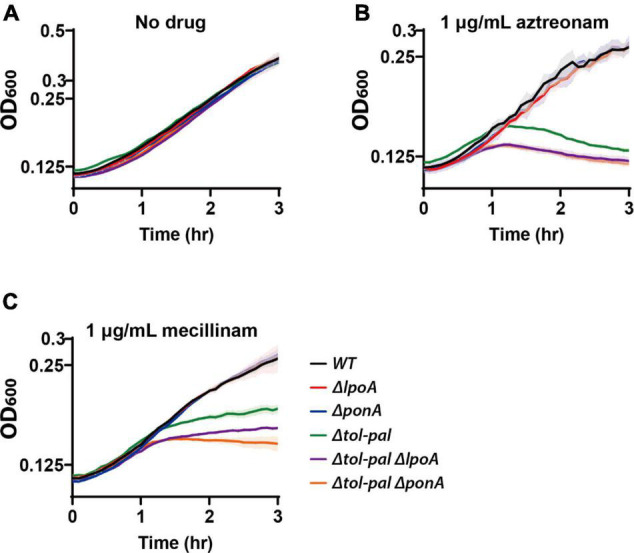
Inactivation of the PBP1a-LpoA system aggravates the defects of the *tol-pal* mutant in maintaining cell integrity. MG1655 (wild type), WJ1 (Δ*ponA*), SP37 (Δ*lpoA*), SP23 (Δ*tol-pal*), SP138 (Δ*tol-pal*Δ*ponA*), and SP139 (Δ*tol-pal*Δ*lpoA*) strains were grown overnight in LB at 30°C. The overnight cultures were diluted to an OD_600_ of 0.02 in LB and grown to an OD_600_ between 0.2 and 0.3 at 30°C. The exponential cultures were diluted to an OD_600_ of 0.1 in plain LB **(A)** or LB containing either aztreonam **(B)** or mecillinam **(C)** at a final concentration of 1 μg/ml and grown at 30°C. The optical density of the cultures was measured in triplicate in a 96-well microtiter plate at 30°C for 3 h with agitation after beta lactam treatment.

The synthetic phenotype between inactivation of the Tol-Pal and PBP1b-LpoB systems prompted us to test if inactivation of the PBP1a-LpoA system also causes a synthetic phenotype with the *tol-pal* mutation. However, unlike inactivation of the PBP1b-LpoB system, mutation of the PBP1a-LpoA system in the *tol-pal* mutant did not cause an obvious defect in maintaining cell integrity in normal growth conditions (Figure 6A). This result was not surprising because mutation of the PBP1a-LpoA system does not usually cause an obvious defect in *E. coli* except for the synthetic lethality with the inactivation of the PBP1b-LpoB system (Supplementary Figure 2; Mueller et al., 2019; Vigouroux et al., 2020). Next, we tested if inactivation of the PBP1a-LpoA system affects the lysis phenotype of the Δ*tol-pal* strain upon beta lactam treatment. Interestingly, lysis of the *tol-pal* mutant upon treatment of aztreonam or mecillinam was further accelerated by mutation of the PBP1a-LpoA system, indicating that the synthetic phenotype with the *tol-pal* mutation is not specific to inactivation of the PBP1b-LpoB system, but can also be observed with inactivation of the PBP1a-LpoA system albeit at a small extent (Figures 6B,C).

### Accelerated Lysis of the Tol-Pal Mutant Upon Mecillinam Treatment Is Suppressed by Production of an LpoB-Bypass PBP1b

**FIGURE 7:**
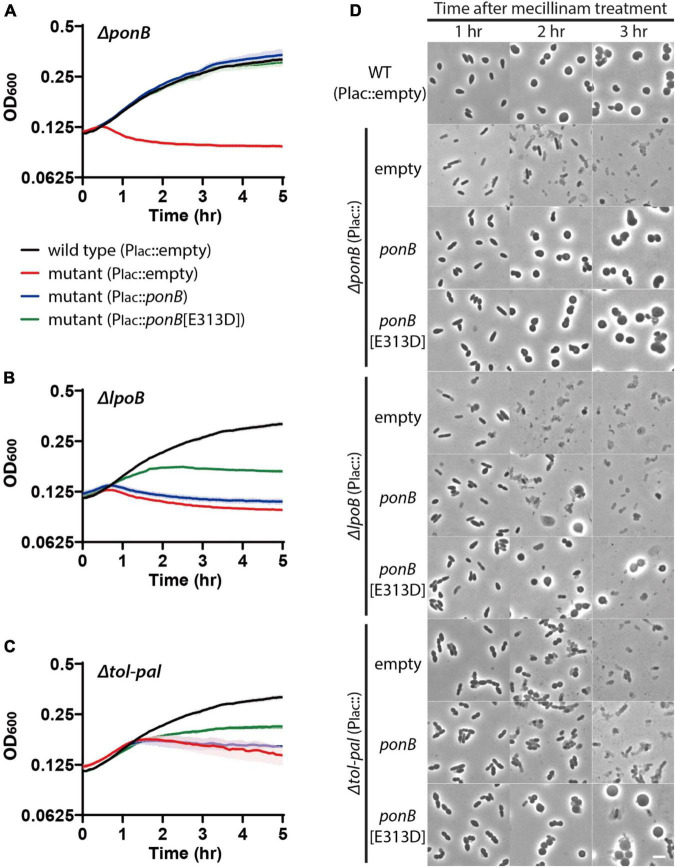
Accelerated lysis of the *tol-pal* mutant upon mecillinam treatment is partially suppressed by production of LpoB-bypass PBP1b. **(A–C)** Lysis curves of Δ*ponB*, Δ*lpoB*, and Δ*tol-pal* strains expressing wild-type *ponB* or the LpoB-bypass *ponB* allele upon treatment with 1 μg/ml mecillinam. MG1655 (attHKpMT116, P_*lac*_:empty), WJ2[Δ*ponB*](attHKpMT116)], WJ2 (attHKpSP23, P_*lac*_:*ponB*), and WJ2 [attHKpSP24, P_*lac*_:*ponB*(E313D)] strains **(A)** were grown overnight in LB at 30°C. The overnight cultures were diluted to an OD_600_ of 0.02 in LB supplemented with 1 mM IPTG to induce expression of *ponB* alleles and grown to an OD_600_ between 0.2 and 0.3 at 30°C. The cultures were then diluted in LB supplemented with 1 mM IPTG and 1 μg/ml mecillinam to an OD_600_ of 0.1 and the optical density of each culture was measured as described in Figure 1. The lysis phenotype of Δ*lpoB* **(B)** and Δ*tol-pal* **(C)** strain derivatives expressing either the *ponB* or *ponB*[E313D] allele was also monitored using the same procedure. **(D)** Strains used for observing the lysis pattern in panels **(A–C)** were incubated in test tubes at 30°C after dilution in LB supplemented with 1 mM IPTG and 1 μg/ml mecillinam. Aliquots were taken at 1–3 h after mecillinam treatment, and the cell shape and lysis phenotype of cells in each culture were observed using phase contrast optics. The scale bar represents 5 μm.

The above results suggested that the Tol-Pal system is required for maintaining cell integrity during elongation. Because all other genes identified as required for maintaining cell integrity in our Tn-seq screen are either PG synthetic or remodeling enzymes, we suspected that the Tol-Pal system would also play a role in PG assembly during cell elongation. The similarity of the lysis phenotype of the *tol-pal* and *ponB/lpoB* mutants upon beta-lactam treatment suggested that the functions of the two systems might be related. We hypothesized that the Tol-Pal system is required for the optimal activity of aPBP-Lpo systems during elongation. If so, the accelerated lysis of the *tol-pal* mutant upon beta-lactam treatment might be caused by inefficient activity of the aPBP-Lpo systems, especially the PBP1b-LpoB system. In addition, the synthetic phenotypes observed upon simultaneous inactivation of an aPBP-Lpo system and the Tol-Pal system might occur because the remaining aPBP-Lpo system does not function optimally in the absence of the Tol-Pal system. To examine our hypothesis, we tested if the accelerated lysis of the *tol-pal* mutant upon beta-lactam treatment is suppressed by enhancing aPBP-Lpo system activity. At first, we tested the effect of increasing expression of aPBPs and their activators. However, overproduction of aPBPs along with their cognate Lpo factors did not suppress the accelerated lysis of the *tol-pal* mutant (Supplementary Figure 3). We reasoned that simple overproduction of aPBPs and Lpo factors might not suppress the accelerated lysis of the *tol-pal* mutant if the Tol-Pal system is required for the efficient activation of aPBPs by Lpo factors. If so, expression of aPBPs that do not require activation by Lpo factors might suppress the Tol-Pal defect. Thus, we examined the effects of enhancing aPBP activity by expressing Lpo factor-bypass aPBPs that do not require Lpo factors for their PG synthetic activity (Markovski et al., 2016; Sardis et al., 2021). To our amazement, expression of *ponB*[E313D] encoding an LpoB-bypass PBP1b protein significantly suppressed the accelerated lysis of the *tol-pal* mutant as well as that of the *lpoB* mutant upon mecillinam treatment, while expression of wild-type *ponB* did not suppress the Tol-Pal defect (Figure 7 and Supplementary Figure 4). Importantly, suppression of the Tol-Pal defect by *ponB*[E313D] expression did not appear to be due to PBP1b[E313D] being more active than wild-type PBP1b. The *ponB*[E313D] allele was less efficient than wild-type *ponB* in suppressing the accelerated lysis of the Δ*ponB* strain upon beta lactam treatment (Supplementary Figure 5). These results suggested that the defect of the *tol-pal* mutant in maintaining cell integrity during elongation might be at least partially due to inefficient aPBP activity, and the Tol-Pal system might be involved in facilitating the activation of PBP1b by LpoB in the elongating cells.

## Discussion

In addition to serving as important therapeutic agents, beta lactam antibiotics have been utilized as useful reagents for studying bacterial cell wall assembly. In this study, we developed a Tn-seq-based genetic screen for identifying factors that play important roles in maintaining cell integrity upon beta lactam treatment. This screen revealed an unanticipated role of the Tol-Pal system in maintaining cell integrity during elongation. The Tol-Pal system has been thought to function mainly at the division sites because the *tol-pal* mutants exhibit pleiotropic phenotypes related to cell division and the Tol-Pal system components localize to the division sites (Gerding et al., 2007; Szczepaniak et al., 2020a; Yakhnina and Bernhardt, 2020). We found that mutants of the Tol-Pal system exhibited a severe defect in maintaining cell integrity upon treatment of mecillinam that specifically targets the Rod system. Further analyses of the Tol-Pal defect suggested that the Tol-Pal system plays an important role in maintaining cell integrity during cell elongation as well as during division. We also found that the accelerated lysis of the *tol-pal* mutant upon mecillinam treatment is partially suppressed by producing a LpoB-bypass PBP1b, indicating that the Tol-Pal system is related to aPBP-Lpo system activity during cell elongation.

Besides our Tn-seq data, the genetic relationship between the Tol-Pal system and the PBP1b-LpoB system was also hinted at by a chemical genomics study on *E. coli* (Nichols et al., 2011). Regarding the genetic relationship, it was previously proposed that the Tol-Pal system regulates PBP1b activity *via* direct interaction of TolA and CpoB with PBP1b at the division sites (Gray et al., 2015; Egan et al., 2018). An alternative but not mutually exclusive explanation for the genetic relationship between the two systems was that the Tol-Pal complexes enhance aPBP-mediated PG synthesis by facilitating the interaction between aPBPs in the IM and Lpo factors in the OM. In Gram-negative bacteria, the PG synthetic activity of aPBPs in the IM is activated by their cognate Lpo factors in the OM (Paradis-Bleau et al., 2010; Typas et al., 2010). Because the PG layer is located between the two membranes, the interaction between aPBPs and their Lpo activators is likely to be inhibited by a tightly woven PG matrix, while it would be facilitated by gaps in the PG (Typas et al., 2010; Lai et al., 2017). Thus, the spatial arrangement of aPBPs and their activators seems to constitute an ideal system for enhancing aPBP activity where PG synthetic activity is required for maintaining the integrity of the cell wall. Because the interaction between the IM and OM components of the Tol-Pal system is also thought to be facilitated by gaps in the PG (Szczepaniak et al., 2020a,b), the Tol-Pal transenvelope complexes are likely to form where aPBP activity is required for maintaining cell integrity. Thus, promotion of aPBP activity by the Tol-Pal transenvelope complexes would further ensure that aPBP activity is enhanced where it is required most for maintaining cell wall integrity.

Suppression of the Tol-Pal defect in maintaining cell integrity upon mecillinam treatment by production of LpoB-bypass PBP1b, but not by wild-type PBP1b, appears to be consistent with the idea that the Tol-Pal system facilitates LpoB-mediated activation of PBP1b activity. Although we did not observe the suppression of the Tol-Pal defect by production of LpoA-bypass PBP1a, we suspect that the Tol-Pal system might also be involved in facilitating the activation of PBP1a by LpoA. We reason that synthetic lysis caused by PBP1b depletion and the *tol-pal* mutation might occur because the remaining aPBP-Lpo system, PBP1a-LpoA, cannot function efficiently in the absence of the Tol-Pal system. Suppression of the Tol-Pal defect by expression of LpoA-bypass PBP1a might not be observed because PBP1a activity is inherently much weaker than PBP1b activity *in E. coli*. Following the same line of reasoning, inactivation of the PBP1a-LpoA system in the *tol-pal* mutant might not cause an obvious phenotype under normal growth conditions because the PBP1b-LpoB system alone manages to maintain cell wall integrity even in the absence of the Tol-Pal system. Further acceleration of Tol-Pal lysis upon beta lactam treatment by an additional mutation of the PBP1a-LpoA system might occur because the PBP1b-LpoB system does not function optimally in the absence of the Tol-Pal system.

In this report, we developed a Tn-seq-based screen to identify genes that play important roles in maintaining cell integrity upon beta-lactam treatment. From this genetic screen, we identified an unanticipated role of the Tol-Pal system during cell wall elongation. Our results also suggest that the Tol-Pal system is required for the optimal PG synthetic activity of the aPBP-Lpo factor systems. We expect that this study can be adapted to develop other approaches involving transposon insertion libraries and cell envelope-targeting drugs for identification of genes important for cell envelope biogenesis.

## Data Availability Statement

The original contributions presented in the study are included in the article/Supplementary Material, further inquiries can be directed to the corresponding author.

## Author Contributions

HC and SP designed research, performed research, and analyzed data. HC wrote the manuscript. Both authors contributed to the article and approved the submitted version.

## Conflict of Interest

The authors declare that the research was conducted in the absence of any commercial or financial relationships that could be construed as a potential conflict of interest.

## Publisher’s Note

All claims expressed in this article are solely those of the authors and do not necessarily represent those of their affiliated organizations, or those of the publisher, the editors and the reviewers. Any product that may be evaluated in this article, or claim that may be made by its manufacturer, is not guaranteed or endorsed by the publisher.
