# Supplementary material for: The Tol-Pal System Plays an Important Role in Maintaining Cell Integrity During Elongation in Escherichia coli
Source: Front Microbiol. 2022 May 3;13:891926. doi: 10.3389/fmicb.2022.891926 (PMC9111525; doi:10.3389/fmicb.2022.891926)
Supplement: Supplementary file 1 [file Data_Sheet_1.docx]

**SUPPLEMENTARY MATERIAL FOR:**

**The Tol-Pal system plays an important role in maintaining cell integrity during elongation in *Escherichia coli*.**

Sohee Park^1^ and Hongbaek Cho^1^*

^1^Department of Biological Sciences, College of Natural Sciences, Sungkyunkwan University,

*To whom correspondence should be addressed.

Hongbaek Cho, Ph.D.

Sungkyunkwan University

Department of Biological Sciences

Suwon 16419, Republic of Korea

e-mail: [hongbaek@skku.edu](mailto:hongbaek@skku.edu)


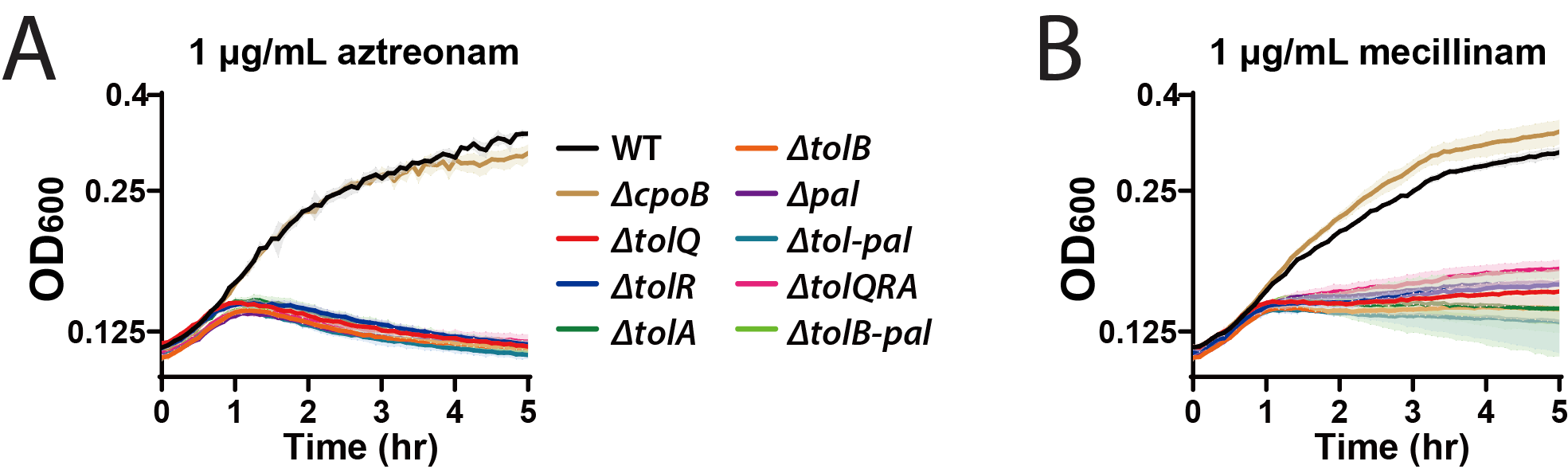


**Supplementary Figure 1. Individual mutation of the Tol-Pal system genes, except for *cpoB*, causes a defect in maintaining cell integrity similar to that caused by complete deletion of the *tol-pal* locus.** MG1655, SP10 (*Δtol-pal*), SP47(Δ*tolB-pal*), SP48(Δ*tolQRA*), SP49 (*ΔcpoB*), SP60 (*Δpal*), SP83 (*ΔtolA*), SP84 (*ΔtolB*), SP166 (*ΔtolQ*) and SP165 (*ΔtolR*) strains were grown overnight in LB at 37 °C. The overnight cultures were diluted to an OD_600_ of 0.02 in LB and grown to an OD_600_ between 0.2 and 0.3 at 37 °C. The exponential cultures were diluted to an OD_600_ of 0.1 in LB containing either aztreonam (A) or mecillinam (B) at a final concentration of 1 μg/ml. The optical density of the cultures was measured in triplicate while being incubated in a plate reader for 5 hours at 30 °C with agitation after beta lactam treatment. The solid lines represent the means and the shades standard deviations.

**
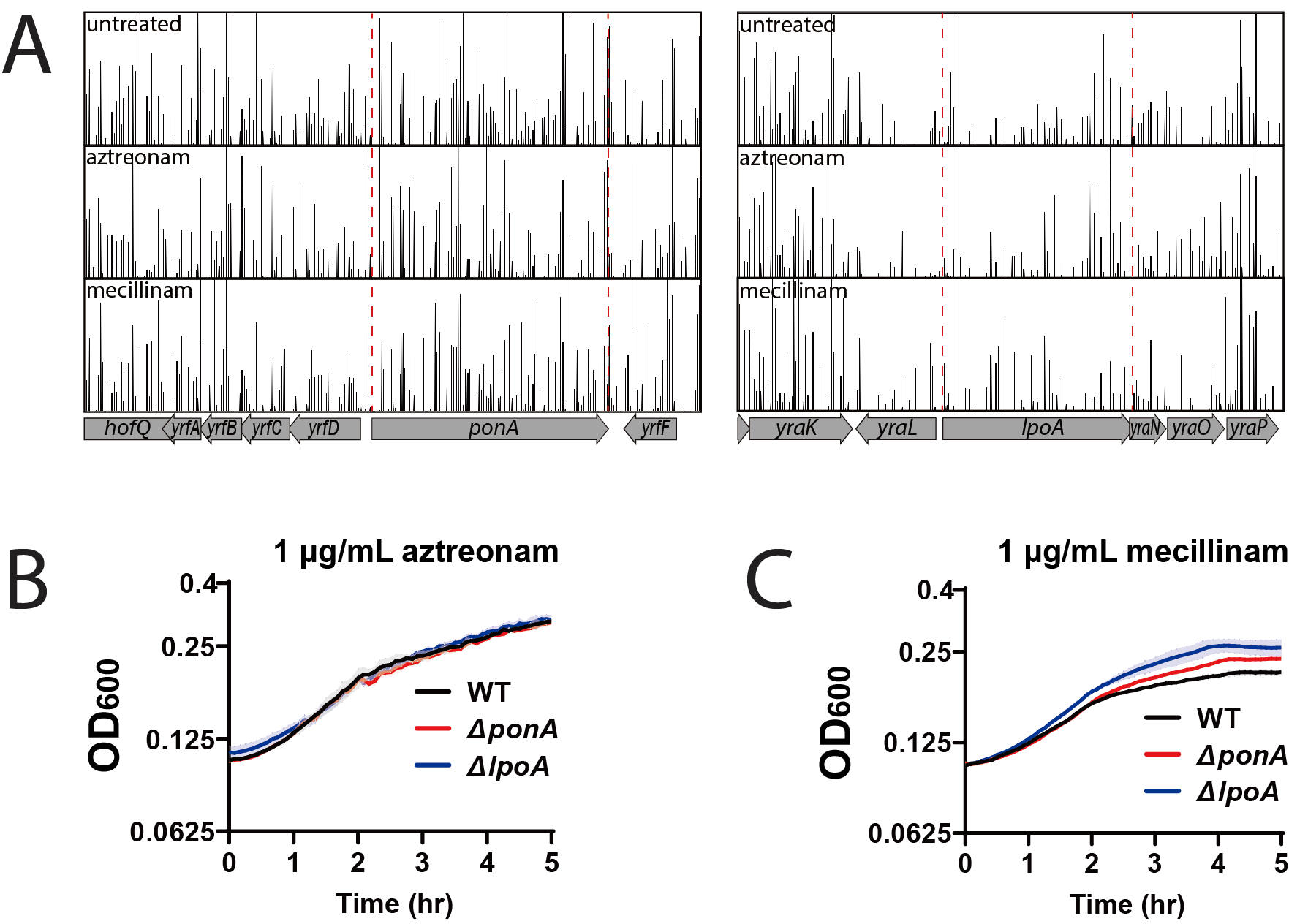
**

**Supplementary Figure 2. Inactivation of the PBP1a-LpoA system does not cause a defect in maintenance of cell integrity upon beta lactam treatment.** (A) Transposon insertion profiles of the genomic region near the *ponA* and *lpoA* genes encoding PBP1a and LpoA, respectively. (B-C) MG1655 (wild type), WJ1 (*ΔponA*), and SP37 (*ΔlpoA*) strains were grown overnight in LB at 30 °C. The overnight cultures were diluted to an OD_600_ of 0.02 in LB and grown at 30 °C to an OD_600_ between 0.2 and 0.3. The resulting exponential cultures were diluted to an OD_600_ of 0.1 in LB containing aztreonam (B) or mecillinam (C) at a final concentration of 1 μg/ml. The optical density of the cultures was measured in the same way as described for Supplementary Figure 1.


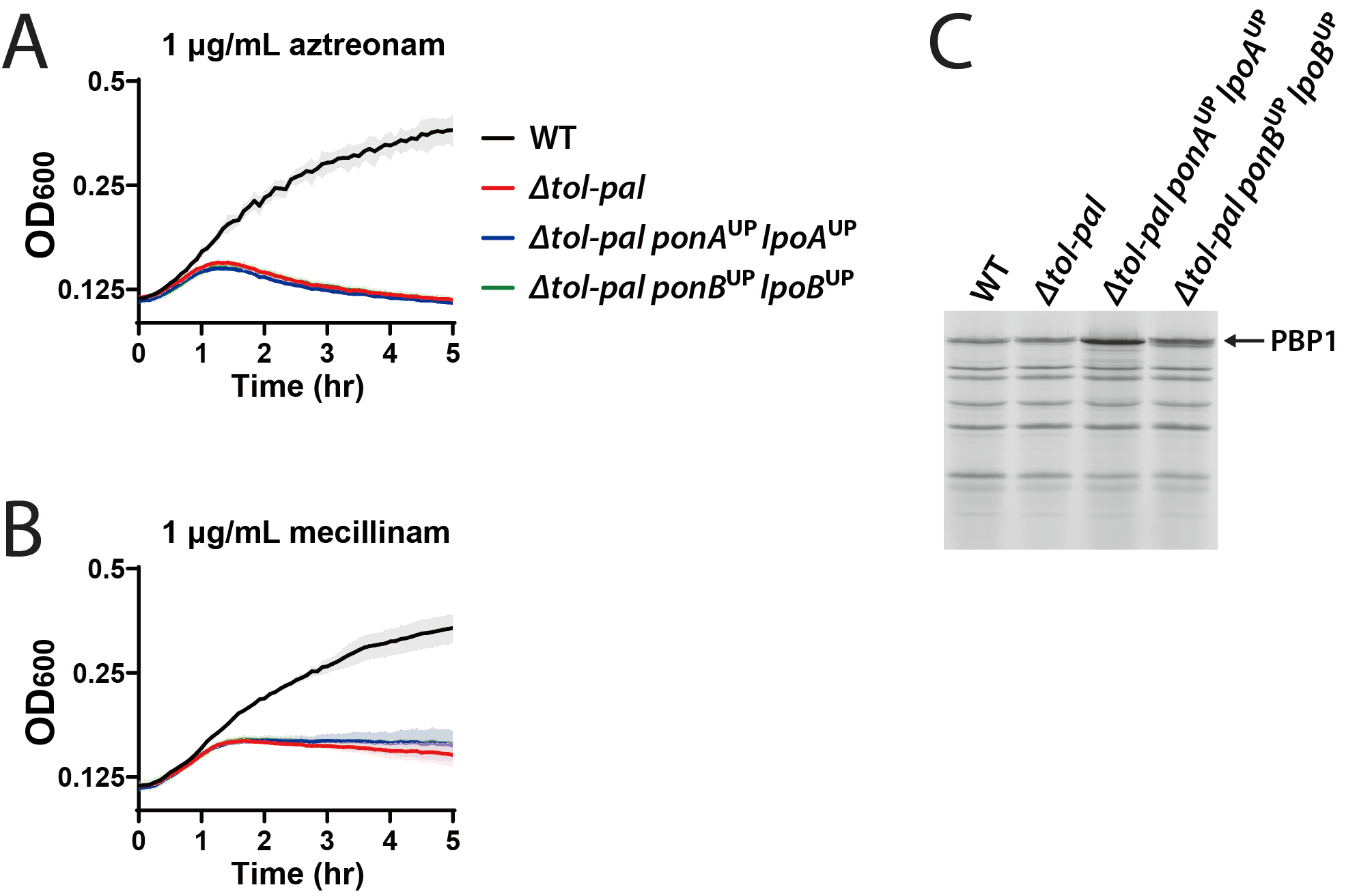


**Supplementary Figure 3. Overexpression of aPBP-Lpo factor pairs does not suppress the accelerated lysis of a *Δtol-pal* strain** MG1655(attHKpMT116, P_lac_::empty)(attλpSP30, P_lac_::empty), SP10[*Δtol-pal*](attHKpMT116) (attλpSP30), SP10(attHKpSP33, P_lac_::*ponA*)(attλpSP34, P_lac_::*lpoA*) and SP10(attHKpSP23, P_lac_::*ponB*)(attλpSP31, P_lac_::*lpoB*) strains were grown overnight in LB at 30 °C. The overnight cultures were diluted to an OD_600_ of 0.02 in 1mM IPTG LB and grown to an OD_600_ between 0.2 and 0.3. The exponential cultures were diluted to an OD_600_ of 0.1 in LB containing either aztreonam (A) or mecillinam (B) at a final concentration of 1 μg/ml. The optical density of the cultures was measured in the same way as described for Supplementary Figure 1. (C) Bocillin assay to visualize the level of PBP1a and PBP1b in the strains used for testing the effect of overexpressing aPBPs and Lpo factors. Cultures of the strains used in (A) and (B) were were grown to the exponential phase (OD_600_ = 0.5) in LB at 30 °C and labelled with Bocillin as described in Figure 3.


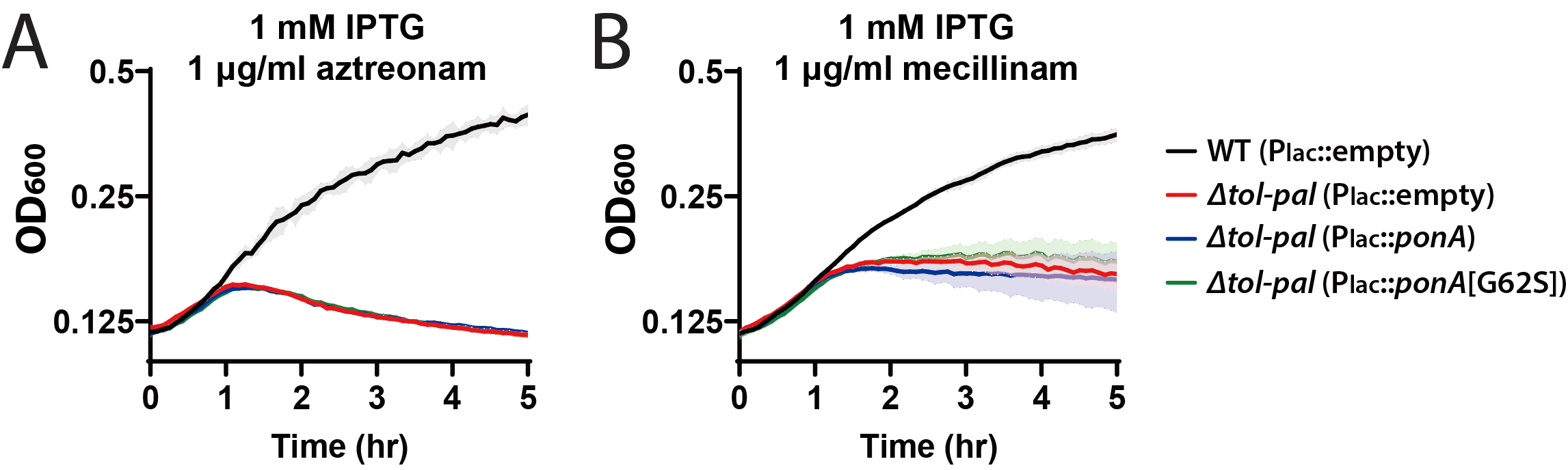


**Supplementary Figure 4. Accelerated lysis of a *Δtol-pal* strain upon beta lactam treatment is not suppressed by expression of an LpoA-bypass *ponA* allele.** MG1655 and SP10 (*Δtol-pal*) strains containing the expression constructs (attHKpMT116, P_lac_::empty), (attHKpSP33, P_lac_::*ponA*), or (attHKpSP46, P_lac_::*ponA*[G62S]) were grown overnight in LB at 30 °C. The overnight cultures were diluted to an OD_600_ of 0.02 in LB supplemented with 1 mM IPTG and grown to an OD_600_ between 0.2 and 0.3 to induce the expression of *ponA* or *ponA*[G62S] alleles. The resulting cultures were then diluted to an OD_600_ of 0.1 in LB supplemented with 1mM IPTG and a final concentration of 1 μg/ml aztreonam (A) or 1 μg/ml mecillinam (B). The optical density of the cultures was measured in the same way as described for Supplementary Figure 1.


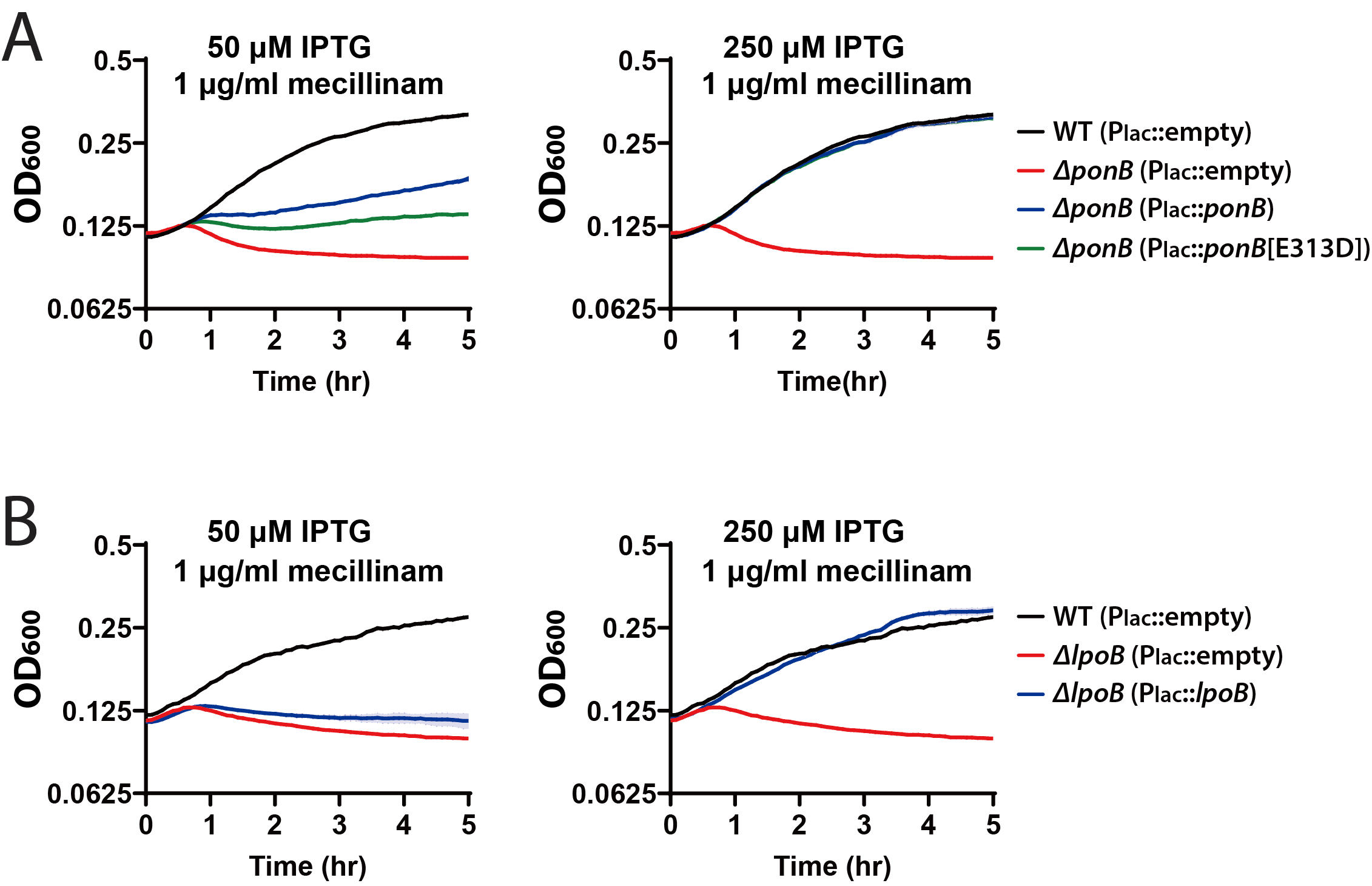


**Supplementary Figure 5. Complementation of the defects of the *ΔponB* and *ΔlpoB* strains in maintaining cell integrity upon mecillinam treatment by expression of *ponB* and *lpoB* alleles.** (A) MG1655 and WJ2 (*ΔponB*) strains containing the expression constructs (attHKpMT116, P_lac_::empty), (attHKpSP23, P_lac_::*ponB*), or (attHKpSP24, P_lac_::*ponB*[E313D]) were grown overnight in LB at 30 °C. The overnight cultures were diluted to an OD_600_ of 0.02 in LB supplemented with the indicated concentrations of IPTG and grown to an OD_600_ between 0.2 and 0.3 to induce the expression of *ponB* or *lpoB* alleles. The resulting cultures were then diluted to an OD_600_ of 0.1 in LB supplemented with a final concentration of 1 μg/ml mecillinam and the indicated concentrations of IPTG. The optical density of the cultures was measured in the same way as described for Supplementary Figure 1. (B) Complementation of the *lpoB* mutation was tested with MG1655 and SP38 (*ΔlpoB*) strains containing the expression constructs (attλpSP30, P_lac_::empty) or (attλpSP31, P_lac_::*lpoB*) using the same procedure.

**Supplementary Table 1. Strains used in this study**

| Strain | Genotype | Source/  Reference |
| --- | --- | --- |
| MG1655 | *rph1 ilvG rfb-50* | Lab collection |
| TB10 | *rph1 ilvG rfb-50 λ*Δ*cro-bio nadA*::*Tn10* | (Johnson et al., 2004) |
| TB28 | *rph1 ilvG rfb-50* Δ*lacIZYA*<>*frt* | (Bernhardt and Boer, 2004) |
| DH5α(*λpir*) | F- *endA1 hsdR17* (*r^-^m^+^*) *supE44 thi-1 recA1 gyrA relA1* Δ(*lacZYA-argF*)*_u189_ Φ80_lac_Z*Δ*M15 λpir* | (Pal et al., 2005) |
| TU115 | *rph1 ilvG rfb-50* Δ*lacIZYA<>frt* Δ*ponA::aph* | (Paradis-Bleau et al., 2010) |
| TU116 | *rph1 ilvG rfb-50* Δ*lacIZYA<>frt* Δ*ponB::aph* | (Paradis-Bleau et al., 2010) |
| MM8 | *rph1 ilvG rfb-50* Δ*lacIZYA<>frt* Δ*lpoA::aph* | (Paradis-Bleau et al., 2010) |
| CB6 | *rph1 ilvG rfb-50* Δ*lacIZYA<>frt* Δ*lpoB::aph* | (Paradis-Bleau et al., 2010) |
| HC261 | *rph1 ilvG rfb-50* Δ*lacIZYA<>frt zapA-gfpmut2::cat* | (Peters et al., 2011) |
| MM10 | *rph1 ilvG rfb-50* Δ*lacIZYA<>frt* Δ*ponA<>frt aph::*P_ara_::*ponB* | (Paradis-Bleau et al., 2010) |
| MM119 | *rph1 ilvG rfb-50 ponB(E313D) yadC::Tn10* Δ*lpoB<>frt* Δ*ponA<>frt* | (Markovski et al., 2016) |
| TB153 | *rph1 ilvG rfb-50* Δ*lacIZYA<>frt* Δ*mepS::aph* | (Truong et al., 2020) |
| SP8 | *rph1 ilvG rfb-50* Δ*mepS::aph* | This study |
| SP10 | *rph1 ilvG rfb-50* Δ*tol-pal::aph* | This study |
| SP23 | *rph1 ilvG rfb-50* Δ*tol-pal<>frt* | This study |
| SP37 | *rph1 ilvG rfb-50* Δ*lpoA::aph* | This study |
| SP38 | *rph1 ilvG rfb-50* Δ*lpoB::aph* | This study |
| SP47 | *rph1 ilvG rfb-50* Δ*tolB-pal::aph* | This study |
| SP48 | *rph1 ilvG rfb-50* ΔtolQRA::aph | This study |
| SP49 | *rph1 ilvG rfb-50* Δ*cpoB::aph* | This study |
| SP60 | *rph1 ilvG rfb-50* Δ*pal::aph* | This study |
| SP83 | *rph1 ilvG rfb-50* Δ*tolA::aph* | This study |
| SP84 | *rph1 ilvG rfb-50* Δ*tolB::aph* | This study |
| SP97 | *rph1 ilvG rfb-50 zapA-gfpmut2<>frt* | This study |
| SP98 | *rph1 ilvG rfb-50 zapA-gfpmut2<>frt* Δ*tol-pal::aph* | This study |
| SP138 | *rph1 ilvG rfb-50 Δtol-pal<>frt ΔponA::aph* | This study |
| SP139 | *rph1 ilvG rfb-50 Δtol-pal<>frt ΔlpoA::aph* | This study |
| SP165 | *rph1 ilvG rfb-50* Δ*tolR::aph* | This study |
| SP166 | *rph1 ilvG rfb-50* Δ*tolQ::aph* | This study |
| SP178 | *rph1 ilvG rfb-50* Δ*slt::aph* | This study |
| SP199 | *rph1 ilvG rfb-50 zapA-gfpmut2<>frt aph*::P_ara_::*ponB* | This study |
| SP200 | *rph1 ilvG rfb-50 zapA-gfpmut2<>frt* Δ*tol-pal<>frt aph*::P_ara_::*ponB* | This study |
| WJ1 | *rph1 ilvG rfb-50* Δ*ponA::aph* | This study |
| WJ2 | *rph1 ilvG rfb-50* Δ*ponB::aph* | This study |

**Plasmid construction**

pBO51 – The *araC*-P_ara_ promoter of pKD46 was replaced with the *cI857*-P_R_ promoter to express *λred* genes from a strong promoter. The *cI857*-P_R_ promoter was amplified with the primer pair 5’-GCTA*GCGGCCGC*AATCGGG-3’ and 5’-GCTA*GAGCTC*AGGCTACGAATTCATACAACCTC-3’ using pKD3 as a template. The amplified DNA was digested with NotI and SacI and ligated with pKD46 digested with the same restriction enzymes.

pSP6 – The p15A replication origin cut out from pMT77 by digestion with NotI and HindIII was ligated to pHC859 digested with the same enzymes to replace the R6K origin of pHC859.

pSP23-pSP24 – The wild-type *ponB* and *ponB*[E313D] sequences were amplified using MG1655 and MM119 as templates with the primer pair 5’- TGAGCGGATAACAATTCCCC*TCTAGA*GAATATTGCGGAGAAAAAGCATG-3’ and 5’- GATATTATCGTGAGATCGAT*AAGCTT*TTAATTACTACCAAACATATCCTTGATC-3’. The *ponB* alleles were cloned into pMT116 digested with XbaI and HindIII using a SLIC (sequence- and ligation-independent cloning) procedure (Jeong et al, 2012).

pSP30 – The *lac* promoter cut out from pMT116 by digestion with BglII and HindIII was ligated to pTB285 digested with the same enzymes to replace the arabinose-inducible promoter (P_ara_) of pTB285.

pSP31 – To clone *lpoB* into pSP30, *lpoB* was amplified with 5’-GCTA*TCTAGA*TTTGTAAGGGGTGAATCTTGATGA-3’ and 5’-GCTA*AAGCTT*TTATTGCTGCGAAACGGCAC-3’. The amplified DNA was digested with XbaI and HindIII, and ligated with pSP30 digested with the same enzymes to generate pSP31.

pSP33 – The *ponA* gene was amplified using the primer pair 5’ -GCTA*TCTAGA*AACTAAATGGGAAATTTCCAGTGAA-3’ and 5’ -GCTA*AAGCTT*TCAGAACAATTCCTGTGCCTC-3’. The resulting PCR product was digested with XbaI and HindIII and ligated with pMT116 digested with the same enzymes to generate pSP33.

pSP34 – The *lpoA* gene was amplified using the primer pair 5’- GCTA*TCTAGA*AAAATATCACTGGATACATTATGGTAC-3’ and 5’- GCTA*AAGCTT*TTAACTGACGGGGACTACCT-3’. The resulting PCR product was digested with XbaI and HindIII and ligated with pSP30 digested with the same enzymes to generate pSP34.

pSP46 – The *ponA* gene of pSP33 was mutated to *ponA*[G62S] by site-directed mutagenesis using the Q5 site-directed mutagenesis kit (NEB # E0552S) according to the manufacturer’s protocol. The whole pSP33 plasmid sequence was amplified using a primer pair 5’-**TC**TGAGAAACGTCGTATTCCG-3’ and 5’-GTATTGAGCAATCAGCTCGC-3’ with Q5 Hot start Enzyme and the amplified DNA was ligated using the KLD enzyme mix to introduce the *ponA*[G62S] mutation into pSP33. The bases that introduce G62S (GGT>>>TCT) are underlined.

**Supplementary Table 2. Plasmids used in this study**

| Plasmid | Genotype* | Origin | Source/  Reference |
| --- | --- | --- | --- |
| pKD13 | *bla aph* cassette flanked by *frt* sequence | R6K | (Datsenko and Wanner, 2000) |
| pKD46 | *bla* *araC* P_ara_::*λred* | pSC101(*ts*) | (Datsenko and Wanner, 2000) |
| pBO51 | *cat* *cI857* P_R_::*λred* | pSC101(*ts*) | This study |
| pCP20 | *bla cat* *repA*(*ts*) *cI857* P_R_::*flp* | pSC101(*ts*) | (Datsenko and Wanner, 2000) |
| pHC859 | *tetAR attHK022* P_tac_::*sulA* | R6K | (Yunck et al., 2016) |
| pINT-ts | *bla* *repA*(*ts*) *cI857* P_R_::*int^λ^* | pSC101(*ts*) | (Haldimann and Wanner, 2001) |
| pMT116 | *tetAR* attHK022 P_lac_::empty | R6K | (Greene et al., 2018) |
| pMT77 | *cat* P_lac_::empty | p15A | (Tsang et al., 2017) |
| pPR66 | *cat* P_lac_::empty | pBR/colE1 | (Greene et al., 2018) |
| pTB102 | *cat cI857 λ*P_R_::*int^HK022^* | pSC101(*ts*) | (Bernhardt and Boer, 2005) |
| pTB285 | *cat attλ* P_ara_::*empty* |  | (Paradis-Bleau et al., 2010) |
| pSP6 | *tetAR* P_tac_::*sulA* | ColE1 | This study |
| pSP23 | *tetAR attHK022* P_lac_::*ponB* | R6K | This study |
| pSP24 | *tetAR attHK022* P_lac_::*ponB*(E313D) | R6K | This study |
| pSP30 | *cat attλ* P_lac_::empty | R6K | This study |
| pSP31 | *cat attλ* P_lac_::*lpoB* | R6K | This study |
| pSP33 | *tetAR attHK022* P_lac_::*ponA* | R6K | This study |
| pSP34 | *cat attλ* P_lac_::*lpoA* | R6K | This study |
| pSP46 | *tetAR attHK022* P_lac_::*ponA(G62S)* | R6K | This study |

**Supplementary Table 3. PCR primers used for gene deletion**

| Target Gene | Primer 1 | Primer 2 |
| --- | --- | --- |
| *tol-pal* | TGCGCTTCCCAAGTCTATTGTCGCGGAGTTTAAGCAGTGAATTCCGGGGATCCGTCGACC | TTACTGCTCATGCAATTCTCTTAGTAAACCAGTACCGCACGTGTAGGCTGGAGCTGCTTC |
| *tolQ* | TGCGCTTCCCAAGTCTATTGTCGCGGAGTTTAAGCAGTGAATTCCGGGGATCCGTCGACC | CACGCGCTCTGGCCATGGCTTACCCCTTGTTGCTCTCGCTTGTAGGCTGGAGCTGCTTC |
| *tolR* | GGCGTTTACCGTTAGCGAGAGCAACAAGGGGTAAGCCATGATTCCGGGGATCCGTCGACC | GCAAGGGAAACGCAGATGTTTAGATAGGCTGCGTCATTAATGTAGGCTGGAGCTGCTTC |
| *tolA* | GAGCGGGTAACAGGCGAACAGTTTTTGGAAACCGAGAGTGATTCCGGGGATCCGTCGACC | CCGAACAGTCAACATCGCGATTACGGTTTGAAGTCCAATGGTGTAGGCTGGAGCTGCTTC |
| *tolB* | ATTATCGTGGGCCATCGGTCCAGATAAGGGAGATATGATGATTCCGGGGATCCGTCGACC | ACTATTCAATTAATTATTATCACAGATACGGCGACCAGGCTGTAGGCTGGAGCTGCTTC |
| *pal* | TGATAATAATTAATTGAATAGTAAAGGAATCATTGAAATGATTCCGGGGATCCGTCGACC | TTACTGCTCATGCAATTCTCTTAGTAAACCAGTACCGCACGTGTAGGCTGGAGCTGCTTC |
| *cpoB* | AAACCGTCGTGCGGTACTGGTTTACTAAGAGAATTGCATGATTCCGGGGATCCGTCGACC | TTCTGGTCGTGTGTTATGCATTACATCGCGTTCAGACGTTTTGTAGGCTGGAGCTGCTTC |
| *tolB-pal* | ATTATCGTGGGCCATCGGTCCAGATAAGGGAGATATGATGATTCCGGGGATCCGTCGACC | TTACTGCTCATGCAATTCTCTTAGTAAACCAGTACCGCACGTGTAGGCTGGAGCTGCTTC |
| *tolQRA* | TGCGCTTCCCAAGTCTATTGTCGCGGAGTTTAAGCAGTGAATTCCGGGGATCCGTCGACC | CCGAACAGTCAACATCGCGATTACGGTTTGAAGTCCAATGGTGTAGGCTGGAGCTGCTTC |
| *slt* | CGCTGCATTGATGTATTTACACTTAGAGGATGCGCTTGTGATTCCGGGGATCCGTCGACC | CATATCATAAACGTGCGGATCAGTAACGACGTCCCCATTCTGTAGGCTGGAGCTGCTTC |

* Underlined are sequences homologous to chromosomal DNA.

**REFERENCES**

Bernhardt, T. G., and Boer, P. A. J. de (2005). SlmA, a Nucleoid-Associated, FtsZ Binding Protein Required for Blocking Septal Ring Assembly over Chromosomes in E. coli. *Mol Cell* 18, 555–564. doi:10.1016/j.molcel.2005.04.012.

Bernhardt, T. G., and Boer, P. A. J. D. (2004). Screening for synthetic lethal mutants in Escherichia coli and identification of EnvC (YibP) as a periplasmic septal ring factor with murein hydrolase activity. *Mol Microbiol* 52, 1255–1269. doi:10.1111/j.1365-2958.2004.04063.x.

Datsenko, K. A., and Wanner, B. L. (2000). One-step inactivation of chromosomal genes in Escherichia coli K-12 using PCR products. *Proc National Acad Sci* 97, 6640–6645. doi:10.1073/pnas.120163297.

Greene, N. G., Fumeaux, C., and Bernhardt, T. G. (2018). Conserved mechanism of cell-wall synthase regulation revealed by the identification of a new PBP activator in Pseudomonas aeruginosa. *Proc National Acad Sci* 115, 201717925. doi:10.1073/pnas.1717925115.

Haldimann, A., and Wanner, B. L. (2001). Conditional-Replication, Integration, Excision, and Retrieval Plasmid-Host Systems for Gene Structure-Function Studies of Bacteria. *J Bacteriol* 183, 6384–6393. doi:10.1128/jb.183.21.6384-6393.2001.

Johnson, J. E., Lackner, L. L., Hale, C. A., and Boer, P. A. J. de (2004). ZipA Is Required for Targeting of D MinC/DicB, but Not D MinC/MinD, Complexes to Septal Ring Assemblies in Escherichia coli. *J Bacteriol* 186, 2418–2429. doi:10.1128/jb.186.8.2418-2429.2004.

Markovski, M., Bohrhunter, J. L., Lupoli, T. J., Uehara, T., Walker, S., Kahne, D. E., et al. (2016). Cofactor bypass variants reveal a conformational control mechanism governing cell wall polymerase activity. *Proc National Acad Sci* 113, 4788–4793. doi:10.1073/pnas.1524538113.

Pal, D., Venkova-Canova, T., Srivastava, P., and Chattoraj, D. K. (2005). Multipartite Regulation of rctB, the Replication Initiator Gene of Vibrio cholerae Chromosome II. *J Bacteriol* 187, 7167–7175. doi:10.1128/jb.187.21.7167-7175.2005.

Paradis-Bleau, C., Markovski, M., Uehara, T., Lupoli, T. J., Walker, S., Kahne, D. E., et al. (2010). Lipoprotein Cofactors Located in the Outer Membrane Activate Bacterial Cell Wall Polymerases. *Cell* 143, 1110–1120. doi:10.1016/j.cell.2010.11.037.

Peters, N. T., Dinh, T., and Bernhardt, T. G. (2011). A Fail-Safe Mechanism in the Septal Ring Assembly Pathway Generated by the Sequential Recruitment of Cell Separation Amidases and Their Activators. *J Bacteriol* 193, 4973–4983. doi:10.1128/jb.00316-11.

Truong, T. T., Vettiger, A., and Bernhardt, T. G. (2020). Cell division is antagonized by the activity of peptidoglycan endopeptidases that promote cell elongation. *Mol Microbiol* 114, 966–978. doi:10.1111/mmi.14587.

Tsang, M.-J., Yakhnina, A. A., and Bernhardt, T. G. (2017). NlpD links cell wall remodeling and outer membrane invagination during cytokinesis in Escherichia coli. *Plos Genet* 13, e1006888. doi:10.1371/journal.pgen.1006888.

Yunck, R., Cho, H., and Bernhardt, T. G. (2016). Identification of MltG as a potential terminase for peptidoglycan polymerization in bacteria. *Mol Microbiol* 99, 700–718. doi:10.1111/mmi.13258.
